# Physiological and ecological responses of flue-cured tobacco to field chilling stress: insights from metabolomics and proteomics

**DOI:** 10.3389/fpls.2024.1490633

**Published:** 2024-11-25

**Authors:** Kaiyuan Gu, Xinkai Li, Jiaen Su, Yi Chen, Chengwei Yang, Juan Li, Chenggang He, Binbin Hu, Congming Zou

**Affiliations:** ^1^ Yunnan Academy of Tobacco Agricultural Sciences, Kunming, Yunnan, China; ^2^ Yunnan Tobacco Company, Dali, Yunnan, China; ^3^ Yunnan Agricultural University, Kunming, Yunnan, China; ^4^ Yunnan Tobacco Company, Chuxiong, Yunnan, China

**Keywords:** flue-cured tobacco, field chilling stress, physiological response, metabolomics, proteomics

## Abstract

**Introduction:**

Currently, research on tobacco's response to chilling stress is mostly limited to laboratory simulations, where temperature is controlled to study physiological and molecular responses. However, laboratory conditions cannot fully replicate the complex environment of field chilling stress, so conducting research under field conditions is crucial for understanding the multi-level adaptive mechanisms of tobacco to chilling stress in natural environments.

**Methods:**

This study aims to use field trials, starting from physiological responses, combined with proteomics and untargeted metabolomics, to systematically reveal the physiological and biochemical characteristics and key molecular mechanisms of tobacco leaves under chilling stress. It provides new insights into tobacco's adaptation strategies under chilling stress.

**Results:**

The results showed that (1) chilling stress damages the appearance of tobacco leaves, reduces the chlorophyll content, increases H_2_O_2_ and malondialdehyde (MDA) levels in cold-injured tobacco leaves, and damages the plasma membrane system. Although catalase (CAT) activity increases to cope with the accumulation of reactive oxygen species (ROS), the activities of key antioxidant enzymes superoxide dismutase (SOD) and peroxidase (POD) significantly decrease, indicating that the antioxidant system of tobacco leaves fails in environments with sudden temperature drops. (2) Proteomics analysis indicated that 410 differentially expressed proteins were identified in cold-stressed tobacco leaves, with 176 upregulated and 234 downregulated. Tobacco leaves under chilling stress attempt to maintain energy supply and physiological stability by enhancing glycolysis, starch, and sucrose metabolism pathways. Concurrently, chilling stress triggers the expression of proteins related to cell wall reinforcement and antioxidant defense. However, due to impaired ribosomal function, protein synthesis is significantly inhibited, which aggravates damage to photosynthesis and cellular functions. (3) Metabolomics analysis revealed that the differential metabolites in cold-stressed tobacco leaves were mainly enriched in tyrosine metabolism, isoquinoline alkaloid biosynthesis, and fatty acid degradation pathways. This indicates that under chilling stress, tobacco leaves enhance adaptability by regulating energy metabolism, increasing antioxidant capacity, and stabilizing cell membrane structure.

**Conclusions:**

Therefore, under chilling stress, tobacco leaves exhibit complex physiological adaptability through multiple regulatory mechanisms involving proteins and metabolites. The research results provide important insights into the metabolic regulatory mechanisms of tobacco in response to extreme environments and also enhance the theoretical foundation for addressing low-temperature stress in practical production.

## 1 Introduction

Tobacco (*Nicotiana tabacum* L.), being a major economic crop extensively cultivated globally, has its yield and quality largely influenced by environmental conditions (Liu et al., 2023; Zhao et al., 2023; Jia et al., 2024). Chilling injury, particularly low-temperature stress during the growing season, has been widely reported as one of the key environmental factors causing significant damage to tobacco production (He et al., 2020). Low temperatures not only suppress tobacco’s photosynthesis and respiration but also induce a range of complex physiological, biochemical, and molecular responses, which in turn impact tobacco’s growth, development, and final yield and quality (Tang et al., 2020; Li et al., 2021; Falcioni et al., 2024; Wang et al., 2024). Thus, researching the response mechanisms of tobacco under low-temperature stress is crucial for improving its stress resistance and ensuring the stability of tobacco production.

Currently, numerous studies have been conducted domestically and internationally on the response of tobacco plants to low-temperature stress (Gao et al., 2024; Shao et al., 2024). Research indicates that chilling injury inhibits tobacco leaf growth, a characteristic mainly reflected in agronomic traits and the quality of fresh tobacco leaves. Low-temperature stress decreases agronomic traits such as plant height, leaf number, and stem circumference in flue-cured tobacco, thus impacting yield formation (Meng et al., 2006; Wang et al., 2009). The low-temperature stress damages the photosynthetic system, antioxidant enzyme system, and osmotic regulation system of tobacco leaves and reduces leaf quality, key chemical components, and production quality, ultimately exacerbating the occurrence of browning reactions in flue-cured tobacco leaves (Gu et al., 2021). Luo et al. (2022) performed transcriptome data analysis on tobacco varieties with varying low-temperature tolerance following cold treatment. The results indicated that the expression levels of genes associated with environmental stress were significantly higher in low-temperature-tolerant varieties than in low-temperature-sensitive varieties, and they identified the role of *NtCBF2* in tobacco’s response to low-temperature stress. Hu et al. (2022) demonstrated a comprehensive analysis of physiological, biochemical, transcriptomic, and metabolomics responses in tobacco leaves under short-term/long-term cold stress and recovery conditions. They unraveled the molecular mechanisms of tobacco’s response to low-temperature stress and identified 12 key hub genes involved in the process.

At present, research on tobacco’s response to chilling injury is largely restricted to laboratory-simulated environments, where low-temperature stress is induced by controlling temperature to investigate the physiological and molecular responses of tobacco (Zhou et al., 2012; Hu et al., 2022; Luo et al., 2022). However, this research method has certain limitations, as indoor experimental conditions cannot fully simulate the variable environmental factors found in natural field environments. In the field, chilling injury not only is caused by temperature changes but also is closely linked to other environmental factors such as soil moisture, light intensity, and wind speed, making the impact of field chilling injury on tobacco more complex and variable. Thus, studying the response mechanisms of tobacco to chilling injury in real field environments is essential for a thorough understanding of tobacco’s adaptive strategies in natural settings.

We hypothesize that low-temperature stress in field conditions significantly reduces the physiological functions of tobacco by inhibiting photosynthesis, disrupting the antioxidant system, regulating the expression of related proteins, and altering metabolite accumulation, and its effects are further complicated by the interactions with environmental factors. Based on the aforementioned issues, we chose to conduct low-temperature stress research under actual field conditions to more accurately simulate and understand the adaptation and response mechanisms of tobacco to chilling injury in natural environments. This study systematically investigates the effects of chilling injury on tobacco’s physiological functions and its molecular response strategies from the perspectives of physiology, proteomics, and metabolomics. The research aims to enrich our understanding of tobacco’s stress resistance physiology and provide theoretical guidance and practical foundations for managing tobacco’s resistance to low temperatures in production. Through research in real field environments, we hope to provide more effective stress resistance strategies for the tobacco industry to address the challenges posed by global climate change.

## 2 Materials and methods

### 2.1 Experimental materials

The experiment was carried out in Jianji Village, Laojunshan Town, Jianchuan County, Dali Bai Autonomous Prefecture, Yunnan Province (E 99°33′, N 26°31′, altitude 2,565 m). The experimental material was the flue-cured tobacco variety Honghua Dajinyuan, cultivated using floating seedling technology. Transplanting of the seedlings under the film was conducted in the year 2021, with a row spacing of 120 cm × 60 cm. Topping was conducted on June 10, leaving 15–16 leaves after topping. Harvesting and curing of the lower leaves began on July 3 and ended on September 7. The soil type used in the experiment was loam, with a pH of 6.47, organic matter content of 56.19 g/kg, total nitrogen of 2.76 g/kg, total phosphorus of 1.11 g/kg, total potassium of 17.64 g/kg, water-soluble nitrogen of 210.8 mg/kg, available phosphorus of 91.3 mg/kg, and available potassium of 285.5 mg/kg.

### 2.2 Experimental design

The experiment was divided into two treatments: Treatment 1 was inside a plastic greenhouse (CK), where a random plot of tobacco leaves measuring 10 m in length and 5 m in width was selected, and a steel-frame greenhouse was constructed. The top was covered with polyvinyl chloride and polyethylene plastic for insulation, rain protection, and light transmission. The sides of the greenhouse were enclosed with two to three layers of shading net to achieve insulation and wind protection. Protective rows were established around the greenhouse. Treatment 2 was conducted under natural field conditions (Treat), where a plot of the same area as in Treatment 1 was randomly selected, with protective rows set up around it. Both treatments were conducted within the same field block, with 60 flue-cured tobacco plants in each treatment. The greenhouse was installed when cold stress was imminent, ensuring that the growth conditions inside and outside the greenhouse were identical before the cold stress.

A TH12R-EX temperature and humidity recorder (Shenzhen Huahanwei Science and Technology Co., Ltd., Shenzhen, China) was placed in the experimental field to record daily temperature and humidity changes. Additionally, a WH-2310 wireless weather station (Jiaxing Misu Electronic Co., Ltd., Zhejiang, China) was used to record daily information on temperature, precipitation, and wind speed. Irrigation of tobacco plants inside the greenhouse was conducted as necessary, based on the natural precipitation outside the greenhouse.

The experiment personnel observed meteorological data and tobacco leaf phenotypes. Once the temperature dropped and the tobacco leaves exhibited phenotypic changes, samples were taken. All tobacco samples were collected from the upper leaves (the fifth to sixth leaves from the top).

### 2.3 Meteorological data acquisition

A TH12R-EX temperature and humidity recorder was placed in the experimental field to record daily temperature and humidity changes, while a WH-2310 wireless weather station was used to record daily information on temperature, precipitation, and wind speed. Meteorological data from July to August were collected comprehensively using the weather station, temperature and humidity recorder, and local meteorological bureau, specifically including temperature, rainfall, and wind speed, with a focus on analyzing the meteorological data when cold injury appeared in the field tobacco leaves.

### 2.4 Determination of physiological indicators and plastid pigment content

Each treatment had three biological replicates. For each replicate, flue-cured tobacco plants with consistent growth were selected, and the samples were immediately stored in liquid nitrogen after sampling for the determination of physiological indicators and plastid pigment content.

Superoxide dismutase (SOD) activity was measured using the nitro blue tetrazolium (NBT) reduction method (Sadak et al., 2020), and peroxidase (POD) and catalase (CAT) activities were determined using the guaiacol method (Rácz et al., 2018) and ultraviolet spectrophotometry (Ekinci et al., 2020), respectively. The malondialdehyde (MDA) content was detected using the thiobarbituric acid (TBA) method (Chen et al., 2022), and the hydrogen peroxide (H_2_O_2_) content was determined using the titanium sulfate colorimetric method (Brennan and Frenkel, 1977).

#### 2.4.1 Determination of plastid pigments

Chlorophyll and carotenoids in tobacco leaves were measured using the ethanol-spectrophotometer method (Jia et al., 2013). A chlorophyll meter was used to determine the soil plant analysis development (SPAD) values of the tobacco leaves at various sampling stages, with a measurement accuracy within ±1.0 SPAD units. The measurement sites were the middle sections between the leaf margin and midrib, symmetrical to the main vein. Three sites on each half of the leaf were measured, and the average reading was taken as the chlorophyll value.

### 2.5 Data analysis of physiological indicators

The mean and standard deviation were calculated using IBM SPSS Statistics 27.0 software, and an independent samples t-test was used to analyze the significance of differences.

### 2.6 Untargeted metabolomics

#### 2.6.1 Metabolite extraction

Each treatment had six biological replicates, with an appropriate amount of fresh tobacco leaf tissue samples. After sampling, the samples were immediately stored in liquid nitrogen for metabolite analysis. Tobacco leaf samples at a mass of 100 mg were taken from each replicate (non-freeze-dried samples) and ground in liquid nitrogen. The homogenate was resuspended by vortexing with 500 μL of pre-chilled 80% methanol and 0.1% formic acid. The samples were incubated on ice for 5 minutes and then centrifuged at 15,000 rpm for 10 minutes at 4°C. The supernatant was diluted to obtain a final liquid chromatography–mass spectrometry (LC-MS)-grade solution containing 60% methanol. The samples were then transferred to new Eppendorf tubes with 0.22-μm filters and centrifuged again at 15,000 rpm for 10 minutes at 4°C. Finally, the filtrate was injected into the ultra-high-performance liquid chromatography–tandem mass spectrometry (LC-MS/MS) system for analysis. Equal volumes of each experimental sample were mixed to create quality control (QC) samples. The blank samples consisted of a 60% methanol solution containing 0.1% formic acid. The pretreatment of the experimental samples was identical to that of the blank samples. The experiment was conducted by mixing 100 μL of liquid sample with 400 μL of pre-chilled methanol, followed by vortexing.

#### 2.6.2 UHPLC-MS/MS analysis

The LC-MS/MS analysis was conducted using the Exion LC™ AD system (SCIEX, Framingham, MA, USA) and QTRAP^®^ 6500+ mass spectrometer (SCIEX). Samples were injected into a BEH C8 column (100 mm × 2.1 mm, 1.9 μm) and analyzed in positive ion mode with a 30-minute linear gradient at a flow rate of 0.35 mL/min. The elution solvents included A (0.1% formic acid–water) and B (0.1% formic acid–acetonitrile). The solvent gradient was set as follows: 5% B for 1 minute, 5% to 100% B over 24.0 minutes, 100% B for 28.0 minutes, 100% to 5% B at 28.1 minutes, and 5% B for 30 minutes. The QTRAP^®^ 6500+ mass spectrometer operated in positive ion mode with a curtain gas of 35 psi, medium collision gas, ion spray voltage of 5,500 V, temperature of 500°C, and ion source gases 1 and 2 both set at 55.

#### 2.6.3 Data processing and metabolite identification

Based on high-resolution mass spectrometry (HRMS) detection technology, untargeted metabolomics can detect as many molecular feature peaks in the samples as possible. Using the high-quality mzCloud database constructed from standards in combination with the mzVault and Mass List databases, molecular feature peaks were matched and identified, enabling the identification of as many metabolites in the biological system as possible, thus providing a comprehensive reflection of the total metabolite information.

The raw data obtained from the instrument were preprocessed using CD3.1 data processing software. First, simple filtering was performed based on parameters such as retention time and mass-to-charge ratio. For different samples, peak alignment was conducted based on retention time deviation and mass deviation [parts per million (ppm)] to make the identification more accurate. Then, peaks were extracted based on the set ppm, signal-to-noise ratio (S/N), adduct ions, and other information, and the peak area was quantified. Then, the high-resolution MS/MS spectral databases mzCloud and mzVault, along with the Mass List MS1 database, were used for metabolite identification by database search. The specific principle is as follows: the molecular weight of the metabolite is determined based on the mass-to-charge ratio (*m*/*z*) of the parent ion in the MS1 spectrum, and the molecular formula is predicted using mass deviation (ppm) and adduct ion information before matching with the database. The databases containing MS/MS spectra match the actual MS/MS spectra with the fragment ions, collision energies, and other information in the database for each metabolite, thereby achieving secondary identification of metabolites. Metabolites with a coefficient of variance (CV) less than 30% in the QC samples were retained as the final identification results for subsequent analysis.

#### 2.6.4 Metabolomics data analysis

#### Metabolite data statistics and analysis

After converting the data using the metaX software, principal component analysis (PCA) and partial least-squares discriminant analysis (PLS-DA) were performed to obtain the variable importance in projection (VIP) value for each metabolite. In the univariate analysis section, the statistical significance (p-value) and fold change (FC value) of each metabolite in different comparison groups were calculated based on the t-test. Differential metabolites between treatments were screened according to the criteria of VIP > 1, p-value <0.05, and FC ≥ 2 or FC ≤ 0.5. Correlation analysis of differential metabolites (Pearson’s correlation coefficient) was performed using R language, and a p-value <0.05 was considered significant. The screened differential metabolites were annotated using the Kyoto Encyclopedia of Genes and Genomes (KEGG) database (https://www.genome.jp/kegg/pathway.html).

### 2.7 Proteomics

#### 2.7.1 Protein extraction

Each treatment had three biological replicates, with an appropriate amount of fresh tobacco leaf tissue samples. After sampling, the samples were immediately stored in liquid nitrogen for proteomics analysis. After being brought back to the laboratory, an appropriate amount of the sample was ground into powder under liquid nitrogen conditions. Protein lysis buffer [7 M urea/2 M thiourea/4% sodium dodecyl sulfate (SDS)/40 mM Tris–HCl, pH 8.5/1 mM phenylmethylsulfonyl fluoride (PMSF)/2 mM ethylenediaminetetraacetic acid (EDTA)] was added to the sample, mixed well, and incubated on ice for 5 minutes. Dithiothreitol (DTT) was added to a final concentration of 10 mM, sonicated in an ice bath for 15 minutes, and then centrifuged at 13,000 *g*, 4°C for 20 minutes; the supernatant was transferred to a new centrifuge tube. Four times the volume of cold acetone was added to the centrifuge tube and left at −20°C overnight. Centrifugation was conducted to collect the protein precipitate, which was then air-dried. The protein was redissolved in 8 M urea/100 mM tetraethylammonium bromide (TEAB) (pH 8.0) solution, DTT was added to a final concentration of 10 mM, and a reduction reaction was performed in a 56°C water bath for 30 minutes. Then, iodoacetamide (IAM) was added to a final concentration of 55 mM and left in the dark at room temperature for 30 minutes for alkylation. The protein concentration was determined using the Bradford method (Silvério et al., 2012).

#### 2.7.2 Enzymatic hydrolysis and desalination of proteins

An equal amount of protein from each sample was used for trypsin digestion. Protein at a mass of 100 μg was taken for trypsin digestion. The protein solution was diluted five times with 100 mM TEAB, and then trypsin was added at a mass ratio of 1:50 (trypsin) for digestion overnight at 37°C. The digested peptides were desalted using a C18 column and lyophilized under vacuum after desalting.

#### 2.7.3 Isobaric tag for relative and absolute quantitation labeling and fractionation

The peptides were dissolved in 0.5 M TEAB and labeled according to the instructions of the iTRAQ-8plex kit (SCIEX). After labeling, the samples were mixed, and the mixed peptides were fractionated using the Ultimate 3000 HPLC system (Thermo DIONEX, Sunnyvale, CA, USA). The chromatography column used was a Durashell C18 column (5 μm, 100 Å, 4.6 mm × 250 mm). Peptide separation was achieved under alkaline conditions by gradually increasing the acetonitrile (ACN) concentration at a flow rate of 1 mL/min, collecting one tube per minute. A total of 42 fractions were collected and combined into 12 components. The combined fractions were desalted on a Strata-X column and vacuum-dried.

#### 2.7.4 LC-MS/MS analysis

Mass spectrometry analysis was carried out using Thermo’s Q Exactive Plus LC-MS system. Samples were separated using the UltiMate 3000 RSLCnano system with a nano-flow rate. Peptide samples were dissolved in the loading buffer, aspirated by an autosampler, bound to a C18 trapping column (3 μm, 120 Å, 100 μm × 20 mm), and then eluted onto an analytical column (2 μm, 120 Å, 750 μm × 150 mm) for separation. The analytical gradient was established using two mobile phases (mobile phase A, 2% acetonitrile/0.1% formic acid/98% H_2_O, 97% H_2_O; mobile phase B, 98% acetonitrile/0.1% formic acid/2% H_2_O). The flow rate of the liquid phase was set at 300 nL/min. In data-dependent acquisition (DDA) mode for mass spectrometry analysis, each scan cycle included a full MS scan (R = 70 K, AGC = 3e6, max IT = 20 ms, scan range = 350–1,800 *m*/*z*) followed by 15 MS/MS scans (R = 17.5 K, Automatic Gain Control (AGC) = 2e5, max IT = 100 ms). The higher-energy collisional dissociation (HCD) collision energy was set to 28. The quadrupole isolation window was set to 1.6 Da. The dynamic exclusion time for ion repeat collection was set to 35 seconds.

#### 2.7.5 Proteomics PRM verification

Based on the TRAQ results, an LC-MS/MS system was used to perform relative quantification comparisons of 18 randomly selected proteins in tobacco samples. Targeted quantification of the relevant proteins was carried out using mass spectrometry-based parallel reaction monitoring (PRM) technology. After detection through discontinuous deformation analysis (DDA), the identified peptide segments of the target proteins were added to the inclusion list of the mass spectrometry acquisition method, enabling the mass spectrometer to specifically collect data on these particular peptides. Targeted relative quantification analysis was then performed by extracting fragment ion information.

## 3 Results

### 3.1 Analysis of meteorological changes and phenotypic comparison during cold injury of flue-cured tobacco

**Figure 1:**
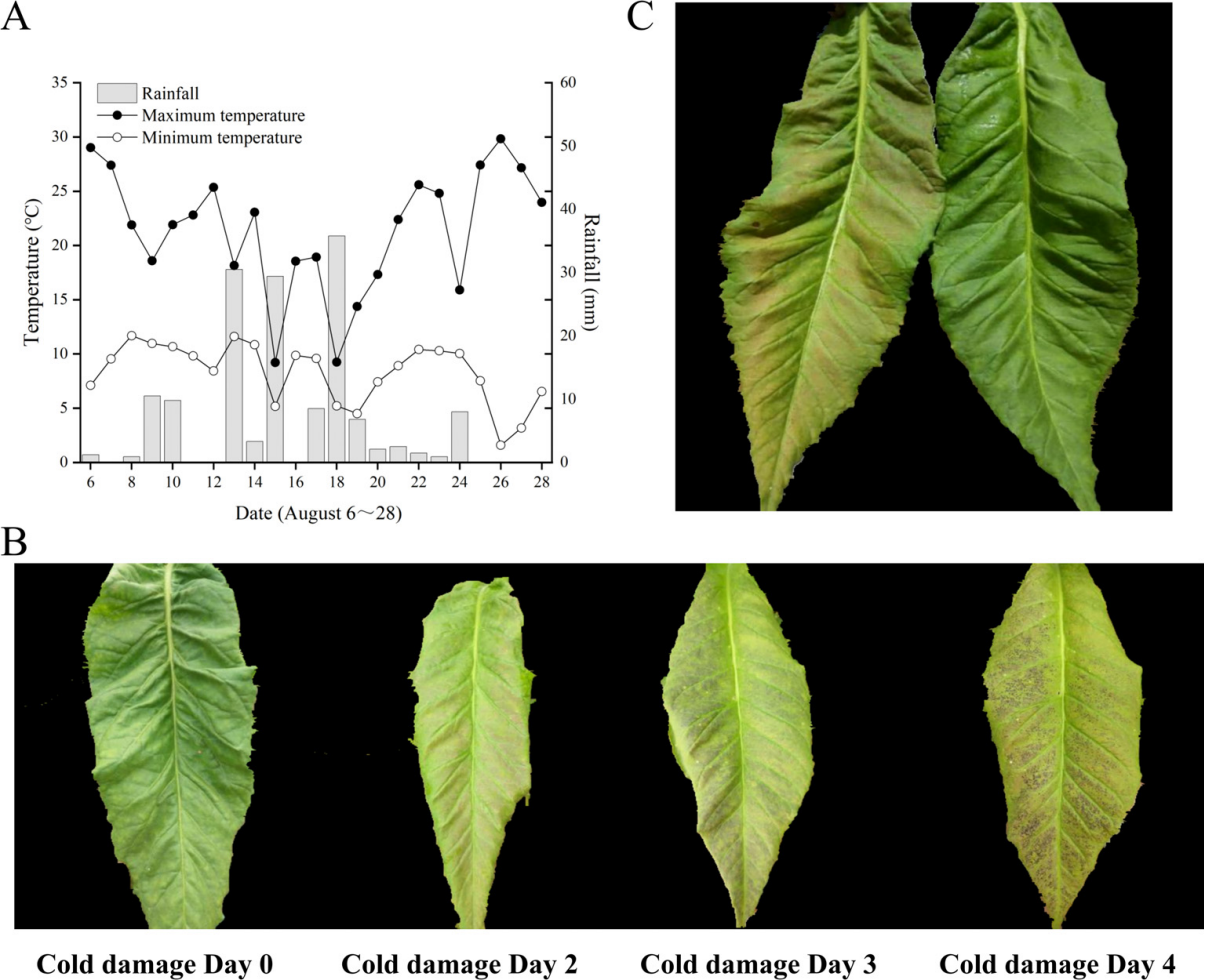
Meteorological data and tobacco leaf phenotypes. **(A)** August meteorological data of Jianji Village experimental site, Laojunshan Town, Jianchuan County. **(B)** Phenotypic changes in chilling-stressed tobacco leaves. **(C)** Treated and control tobacco plant leaves.

As indicated in **Figure 1A**, the temperature and rainfall at the Jianji Village experimental site in Laojunshan Town, Jianchuan County, exhibited considerable fluctuations in August. The daily maximum temperature varied from 14.6°C to 30.8°C, the daily minimum temperature ranged from 8.6°C to 16.6°C, and the daily rainfall ranged from 0 mm to 35.8 mm. On August 26, the greatest diurnal temperature variation was recorded at 22.2°C, but without any rainfall. On August 18, the smallest diurnal temperature variation was 3.1°C, accompanied by 35.8 mm of rainfall. According to the local survey of field chilling stress, a significant temperature drop occurred on August 18, accompanied by heavy rainfall and a small amount of hail. In the following 4–5 days, large-scale chilling stress symptoms appeared in flue-cured tobacco, with the most severe symptoms in the middle and upper leaves. The leaf surface color gradually changed, as shown in **Figure 1B**: the normal tobacco leaf surface was green; after 1 day of chilling stress, the color changed to light pink; after 1–2 days of chilling stress, it changed to dark green; after 3–4 days, the leaf surface turned purple-black.

**Figure 2:**
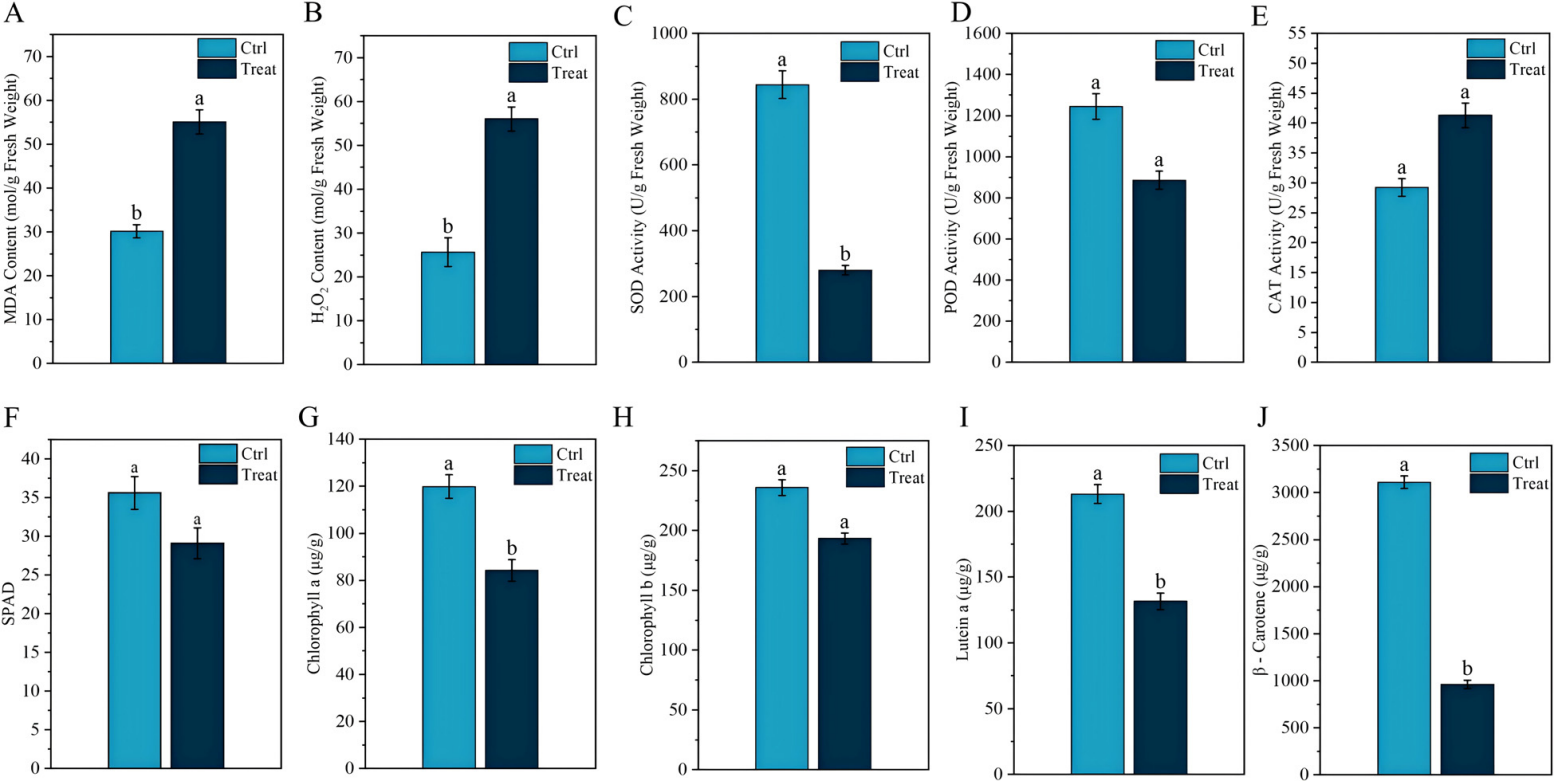
Physiological response of tobacco leaves to chilling stress. Different lowercase letters indicate significant differences when p < 0.05. Data are expressed as mean ± standard error.

On August 19, samples of normal tobacco leaves (CK) and chilling-stressed tobacco leaves (Treat) were collected from the experimental site (**Figure 2C**), with the sampling locations being the fifth to sixth leaves from the bottom to the top. As seen in **Figure 2C**, the normal tobacco leaves on the right showed no obvious symptoms of chilling stress, while the chilling-stressed tobacco leaves on the left clearly showed large pink spots on the leaves, indicating that chilling stress has occurred.

### 3.2 Physiological response of tobacco leaves to chilling stress

MDA, as one of the final products of lipid peroxidation in membranes, can effectively reflect the degree of cell membrane damage. **Figure 2A** shows that the MDA levels in tobacco leaves significantly increase after chilling stress. The transmission of plant stress signals is influenced by changes in H_2_O_2_ content. As shown in **Figure 2B**, the H_2_O_2_ content in tobacco leaves under chilling stress is significantly higher than that in normal tobacco leaves (p < 0.05). Chilling stress also led to a significant decrease in SOD and POD activities in tobacco leaves (p < 0.05) (**Figures 2C, D**), whereas CAT activity significantly increased (p < 0.05) (**Figure 2E**). Additionally, we observed that under chilling stress, the SPAD, chlorophyll *a*, chlorophyll *b*, lutein, and β-carotene values in tobacco leaves were all significantly reduced (p < 0.05) (**Figure 2**).

### 3.3 Metabolomics analysis of tobacco field chilling injury

**Figure 3:**
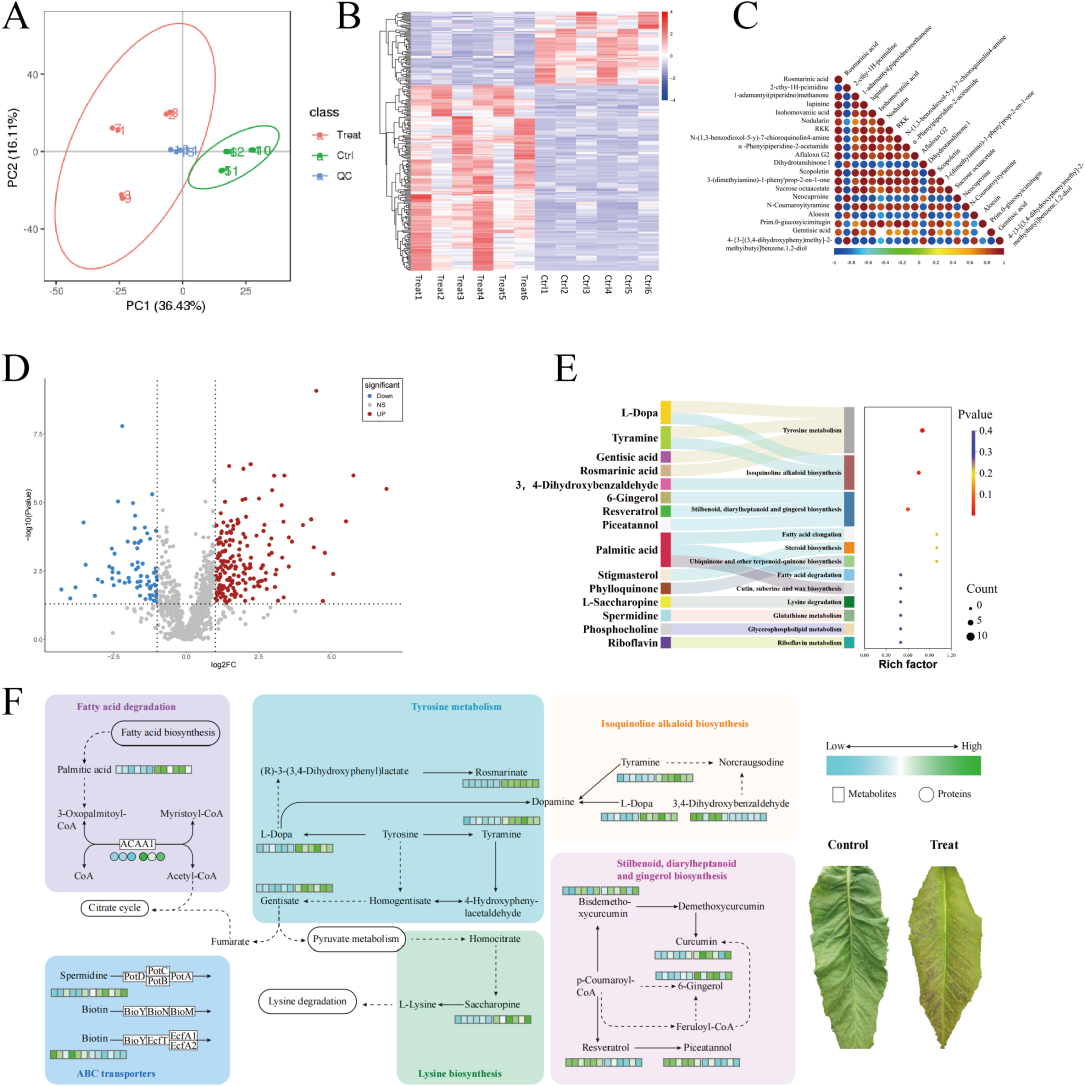
Metabolomics analysis of field chilling injury in flue-cured tobacco. **(A)** Metabolomics analysis of field chilling injury in flue-cured tobacco. **(B)** Cluster heat map of differential metabolites. **(C)** Differential metabolite correlation plot. **(D)** Differential metabolite volcanic map. **(E)** Sankey bubble diagram enriched in differential metabolite KEGG. **(F)** Heat map of differential metabolite pathways. KEGG, Kyoto Encyclopedia of Genes and Genomes.

**Figure 3A** presents the results of the metabolite data identified using PCA, with the first principal component (PC1) scoring 36.43% and the second principal component (PC2) scoring 16.1%. The figure demonstrates that the CK-treated tobacco leaves and the chilling-stressed tobacco leaves were clearly separated, with replicate samples from different treatments closely clustered together, indicating good data reproducibility. Additionally, cluster analysis of differential metabolites was conducted for each of the 12 sample comparison groups in this study. The results, as shown in **Figures 2B, C**, indicate that the biological replicates of the experimental samples are relatively consistent and stable. The selection of differential metabolites was mainly based on three parameters—VIP, FC, and p-value—with thresholds set at VIP > 1.0, FC > 2.0 or FC < 0.5, and p-value <0.05. The differential metabolites selected are shown in **Figure 3D**: a total of 1,035 metabolites were identified in the metabolomics analysis, with 240 differential metabolites identified, of which 172 were upregulated and 68 were downregulated.

As shown in **Figure 3E**, the KEGG enrichment results indicate that the differential metabolites are significantly enriched in three metabolic pathways: Tyrosine metabolism (map00350), Isoquinoline alkaloid biosynthesis (map00950), and Stilbenoid, diarylheptanoid, and gingerol biosynthesis (map00945).

Further analysis revealed that chilling stress significantly upregulated several key metabolites in the Tyrosine metabolism (map00350), Isoquinoline alkaloid biosynthesis (map00950), and Stilbenoid, diarylheptanoid, and gingerol biosynthesis (map00945) pathways in tobacco leaves, including l-Dopa, Tyramine, Gentisic acid, Rosmarinic acid, 6-Gingerol, Resveratrol, and Piceatannol. In addition, we found that in stress resistance-related metabolic pathways, Saccharopine in the Lysine degradation pathway (map00310), Spermidine in the ABC transporter pathway (map02010), and Palmitic acid and ACAA1 protein in the Fatty acid degradation pathway (map01212) were all significantly upregulated, while Biotin was downregulated in the ABC transporter pathway (**Figure 3F**).

### 3.4 Proteomics analysis of tobacco field chilling injury

**Figure 4:**
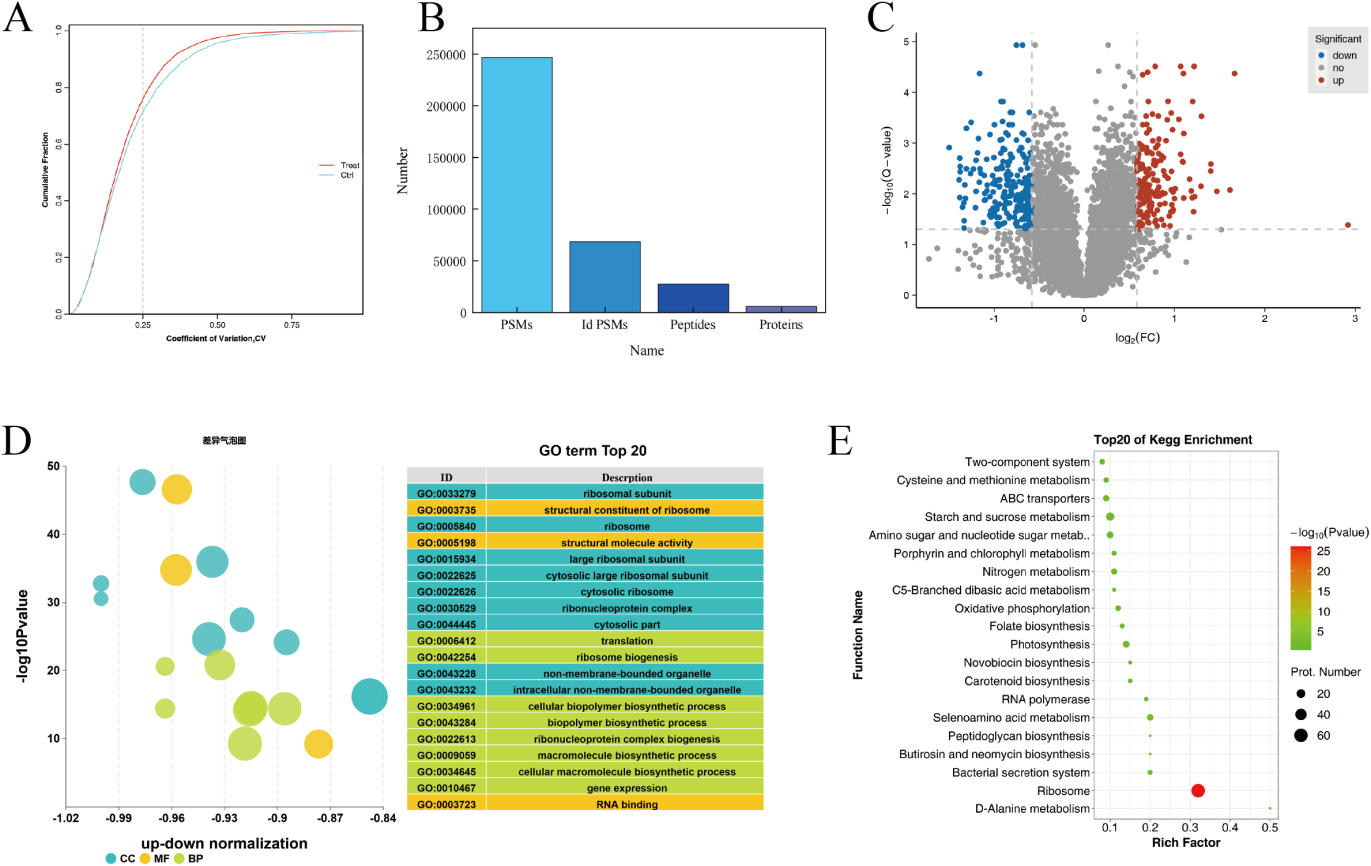
Proteomics analysis of tobacco field chilling injury. **(A)** CV value distribution diagram for different samples. **(B)** Statistical chart of protein identification information. **(C)** Volcanic map of differentially expressed proteins. **(D)** Enrichment analysis of differentially expressed protein via GO. **(E)** Enrichment analysis of differentially expressed protein via KEGG. CV, coefficient of variance; GO, Gene Ontology; KEGG, Kyoto Encyclopedia of Genes and Genomes.

Proteomics analysis was performed on normal and chilling-stressed tobacco leaves. As shown in **Figure 4A**, the reproducibility of the biological replicate sample data was good. A total of 246,819 spectra were obtained, with 68,496 spectra matched, 27,350 peptides identified, and 5,950 proteins identified (**Figure 4B**). On the basis of the entire proteome, the protein data obtained from the biological replicates were combined, and the commonly expressed proteins were used for differential protein screening. A total of 410 commonly differentially expressed proteins were identified between the chilling-stressed and normal tobacco leaves in the comparison group, with 176 upregulated (p ≤ 0.05, fold change >1.5) and 234 downregulated (p ≤ 0.05, fold change <0.67) (**Figure 4C**).

Gene Ontology (GO) functional annotation analysis was performed on the differentially expressed proteins in different groups, focusing on biological processes, cellular components, and molecular functions. **Figure 4D** shows the GO annotation results for the differentially expressed proteins in chilling-stressed and normal tobacco leaves, with the top 20 GO enrichments including eight biological processes, nine cellular components, and three molecular functions. The biological processes were mainly enriched in translation, ribosome biogenesis, cellular biopolymer biosynthetic process, biopolymer biosynthetic process, ribonucleoprotein complex biogenesis, and macromolecule biosynthetic process. The cellular components were mainly enriched in ribosomal subunit, ribosome, large ribosomal subunit, cytosolic large ribosomal subunit, and cytosolic ribosome. The molecular functions were mainly enriched in the structural constituent of ribosome, structural molecule activity, and RNA binding (**Figure 4D**).

KEGG pathway enrichment analysis revealed that the differential proteins were significantly enriched in pathways such as Ribosome (map03010), Starch and sucrose metabolism (map00500), and Glycolysis/Gluconeogenesis (map00010). The upregulated proteins were significantly enriched in metabolic pathways such as Starch and sucrose metabolism (map00500) and Amino sugar and nucleotide sugar metabolism (map00520). The downregulated proteins were significantly enriched in pathways such as Ribosome (map03010) and Photosynthesis (map00195) (**Figure 4E**).

### 3.5 Proteomics PRM verification

**Figure 5:**
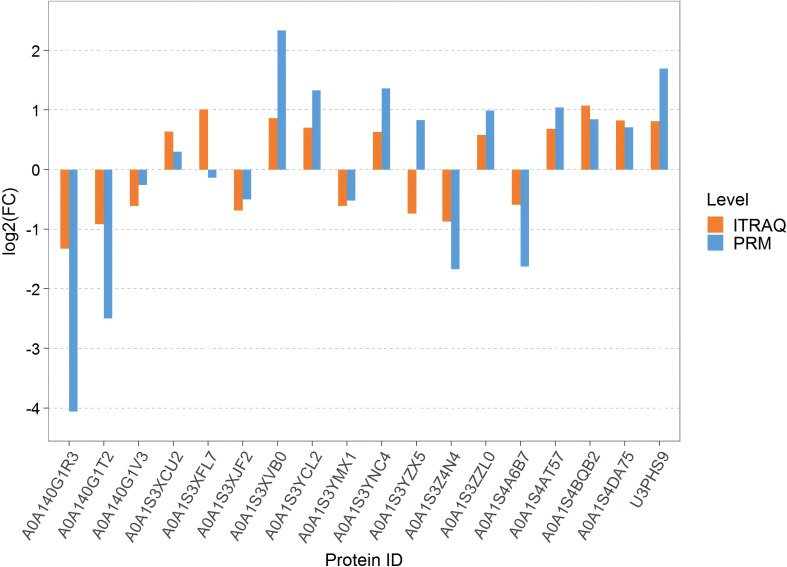
Bar chart of the difference between PRM quantification and ITRAQ. PRM, parallel reaction monitoring; iTRAQ, isobaric tag for relative and absolute quantitation.

To validate the abundance differences of proteins related to chilling-stressed and normal tobacco leaves, 18 important proteins were randomly selected for PRM verification. The comparison of PRM quantification and isobaric tag for relative and absolute quantitation (iTRAQ) for the protein expression differences in the Treatcomparison group is shown in **Figure 5** among them, 16 proteins showed consistent levels of expression changes, while two proteins showed opposite trends. Overall, the results are largely consistent with our iTRAQ-based proteomics quantification results (**Figure 5**).

## 4 Discussion

### 4.1 Physiological response of tobacco leaves to field chilling stress

In summary, the physiological, metabolic, and protein-level responses of tobacco leaves under cold stress form a highly coordinated process, with interactions between these layers working together to ensure the plant’s adaptability to adverse environments. The results of this study reveal the multi-level response mechanisms of tobacco leaves to cold stress, from physiological to metabolic to molecular levels. The measured altitude of the experimental site in this study was 2,565 m, significantly higher than the main tobacco-growing areas in Yunnan. The temperature fluctuations during the curing period were exceptionally intense, with August temperatures ranging from 14.6°C to 30.8°C, making field chilling injury highly likely. The investigation found that on the day of chilling stress (August 18), the temperature difference at the experimental site was 25.4°C. Over the next 4 days, the appearance of the middle and upper tobacco leaves changed from green to light pink, then to dark green, and finally to purple-black. This color change is a visual indicator of the plant’s progression from mild to severe damage due to chilling, reflecting the complex physiological and biochemical changes within the leaves. Research indicates that low-temperature stress causes numerous visible changes in plants, particularly phenotypic alterations, representing the most direct expression of environmental interference. With prolonged exposure to low-temperature stress, plants show visible symptoms such as wilting, slowed growth, and leaf chlorosis (Cheng et al., 2021). Under continued chilling stress, cells may undergo irreversible damage leading to cell death. The rupture of cell membranes and lipid peroxidation reactions are likely the primary causes of the tobacco leaves turning dark green or purple-black (Zhang et al., 2020; Bilska-Kos et al., 2017; Stefanowska et al., 1999).

Research indicates that the level of photosynthetic pigments is closely related to photosynthetic function (Wang et al., 2022; Yin et al., 2022), and it is generally believed that the content of photosynthetic pigments decreases when plants are subjected to stress (Jaleel et al., 2008; Anjum et al., 2017). In this study, after chilling stress, the chlorophyll and carotenoid contents in tobacco leaves significantly decreased compared to the control, indicating that chilling stress directly inhibited the photosynthetic capacity of the tobacco leaves. The reduction in photosynthetic pigments not only affects the efficiency of light absorption and conversion but may also lead to structural damage to the photosynthetic apparatus, which corresponds to the color changes observed in tobacco leaves after chilling stress.

Abiotic stresses such as drought, low temperature, salt stress, and heavy metal pollution significantly inhibit plant growth and development. These adverse conditions can damage or disrupt various metabolic processes within plant cells, leading to a substantial accumulation of reactive oxygen species (ROS) (Choudhury et al., 2017). H_2_O_2_, as a primary form of ROS, can indicate the extent of damage in plants (Hossain et al., 2015). When plants are subjected to stress, lipid peroxidation of cell membranes occurs, leading to increased membrane permeability. MDA, a byproduct of lipid peroxidation, increases in content, reflecting the degree of peroxidation and the intensity of the stress faced by the plant (Orts et al., 2024). Previous studies have found a positive correlation between decreasing temperature and increasing MDA content (Gao et al., 2021). In this study, chilling stress significantly increased the levels of H_2_O_2_ and MDA in tobacco leaves, indicating that the tobacco leaf cells suffered severe oxidative damage, with the structure and function of cell membranes impaired. Although plants typically eliminate excess ROS through their antioxidant system, under the chilling stress in this study, the activities of key antioxidant enzymes such as SOD and POD were significantly reduced, with only CAT activated in an attempt to remove the excess ROS (He et al., 2017). This is contrary to previous studies on the response of antioxidant enzymes in tobacco leaves to chilling stress (Gu et al., 2021), possibly because, under natural conditions, the large and sudden temperature drop caused the tobacco leaves’ antioxidant system to be unable to adapt to the rapidly changing environment in a short time, thereby weakening its ability to eliminate ROS.

### 4.2 Metabolomics analysis of tobacco field chilling injury

Changes at the physiological level are often accompanied by the reorganization of metabolic networks to enhance the plant’s resistance to stress. In this study, chilling stress significantly upregulated several key metabolites in the tyrosine metabolism, isoquinoline alkaloid biosynthesis, and stilbenoid, diarylheptanoid, and gingerol biosynthesis pathways in tobacco leaves, including l-Dopa, Tyramine, Gentisic acid, Rosmarinic acid, 6-Gingerol, Resveratrol, and Piceatannol. The upregulation of these metabolites may reflect a physiological response in tobacco to enhance its resistance to chilling stress by strengthening antioxidant and defense mechanisms. l-Dopa and Tyramine play important roles in the tyrosine metabolism and isoquinoline alkaloid biosynthesis pathways, and their increase may promote the accumulation of antioxidant compounds and defense-related secondary metabolites, enhancing the plant’s ability to cope with oxidative stress (Schenck and Maeda, 2018; Cherubino Ribeiro et al., 2023). Additionally, Gentisic acid and Rosmarinic acid, as phenolic compounds with strong antioxidant capacities, may be upregulated to further protect cells from oxidative damage caused by chilling stress (Cao et al., 2005; Li et al., 2022). The upregulation of polyphenolic compounds such as 6-Gingerol, Resveratrol, and Piceatannol suggests that the plant may mitigate the damage to cell structure and function caused by chilling by enhancing its antioxidant capacity (Stagos, 2019).

Additionally, we found that in stress resistance-related metabolic pathways, Saccharopine in the Lysine degradation pathway, Spermidine in the ABC transporter pathway, and Palmitic acid and *ACAA1* protein in the Fatty acid degradation pathway were significantly upregulated, while Biotin was downregulated in the ABC transporter pathway. These changes suggest that flue-cured tobacco enhances its stress adaptation through various metabolic regulatory strategies under chilling stress. The accumulation of Saccharopine may be related to the carbon skeleton supply and NADH production in the lysine degradation pathway, providing energy support for stress response (Fujioka, 1975). Spermidine, as a polyamine, may help stabilize cell membranes and enhance antioxidant defense when upregulated (Saleethong et al., 2016), while the downregulation of Biotin may be related to the plant’s metabolic reprioritization under low-temperature conditions. Additionally, the upregulation of Palmitic acid and *ACAA1* protein suggests accelerated fatty acid degradation, providing more energy and metabolic intermediates to support the plant’s physiological needs under chilling stress (Guan and Liu, 2020). These results indicate that chilling stress enhances the accumulation of various stress resistance-related metabolites in tobacco leaves by regulating multiple metabolic pathways, thereby improving the plant’s adaptation to low-temperature environments.

### 4.3 Proteomics analysis of tobacco field chilling injury

The accumulation of metabolites depends on protein regulation, and changes in protein expression further modulate the plant’s stress resistance strategies. After tobacco leaves are subjected to chilling injury, energy metabolism plays a crucial role. Insufficient energy supply can lead to physiological imbalances in the tobacco leaves, thereby exacerbating the occurrence of chilling injury. In this study, under chilling stress, proteins in the glycolysis pathway, including hexokinase (*HK*), glyceraldehyde 3-phosphate dehydrogenase (*GAPDH*), and enolase (*ENO*), were significantly upregulated. Similarly, proteins in the Starch and sucrose metabolism pathway, such as hexokinase (*HK*) and glucan endo-1,3-beta-d-glucosidase (*EGLC*), were also significantly upregulated (Ruan, 2014; Poonam et al., 2016). Additionally, the Amino sugar and nucleotide sugar metabolism pathways play a crucial role in plant stress resistance through various mechanisms, including cell wall reinforcement, antioxidant defense, signal regulation, energy support, and immune enhancement (Ma et al., 2021; Ran et al., 2023). The study found that chilling stress activated the upregulation of proteins such as Chitinase (E3.2.1.14) and Hexokinase (HK) in the Amino sugar and nucleotide sugar metabolism pathways in tobacco leaves. This further indicates that the synergistic effects of multiple metabolic pathways help tobacco leaves maintain cell structure integrity and physiological function under chilling conditions, thereby enhancing the plant’s overall stress resistance.

However, under low-temperature stress, 61 proteins in the Ribosome (map03011) pathway, including Small subunit ribosomal protein S10 (RP-S10), Large subunit ribosomal protein L4e (*RPL4*), Large subunit ribosomal protein L2 (*MRPL2*), Large subunit ribosomal protein L14 (*MRPL14*), and Small subunit ribosomal protein S11 (*rpsK*), were downregulated, indicating that chilling injury caused ribosomal instability in tobacco leaves, thereby inhibiting protein synthesis. This is consistent with the findings of Lv et al. (2021) under low-temperature treatment where *Volvariella volvacea* primarily responds to stress by activating the VVO_00750 gene in the ribosome pathway. The ribosome is the central machinery for protein synthesis, and the reduction of ribosomal proteins directly weakens ribosome assembly and stability, thereby reducing overall translation efficiency (Rodnina et al., 2016). This implies that during the chilling process, new proteins cannot be effectively synthesized in tobacco leaves, which in turn hinders the cells’ ability to repair and respond to chilling stress damage.

Furthermore, through additional screening, this study identified differentially abundant proteins enriched in the Photosynthesis pathway in leaves under chilling stress, including *psbC*, *psbF*, and *psbO* of *PSII*; *psaA* and *psaK* of PSI; and *petD*, *petA*, and *petF* of the cytochrome *b*_6_ *f* complex, which is consistent with the findings of Cui et al. (2014) on the differential gene expression profile of tobacco seedlings under low-temperature stress. The downregulation of these proteins indicates that chilling stress inhibited the photosynthetic electron transport chain in tobacco leaves, leading to a decline in photosynthetic efficiency (Peredo and Cardon, 2020). This inhibition is closely related to the reduction in chlorophyll *a* and *b* contents. Chlorophyll *a* and *b* are key pigments in capturing light energy and are involved in energy transfer in photosystems I (PSI) and II (PSII). As these photosynthetic pigments decrease, the light energy capture ability declines, further leading to the downregulation of related proteins in PSII and PSI, ultimately weakening the entire photosynthesis process (Leister and Schneider, 2003; Allakhverdiev et al., 2008). In conclusion, at the protein level, chilling stress intensifies the physiological imbalance and damage in tobacco leaves by disrupting energy metabolism, inhibiting protein synthesis, and impairing photosynthesis in a synergistic manner.

## 5 Conclusion

This research demonstrates that tobacco leaves under field chilling stress employ complex adaptation strategies through multiple regulations at the physiological, metabolic, and protein levels. The oxidative damage, metabolic reorganization, and changes in key protein expression induced by chilling stress reflect the plant’s integrated response capabilities in energy supply, antioxidant defense, and protein synthesis. The synergy between these different levels supports plant survival under stress and reveals the profound impact of chilling stress on essential plant functions. These findings offer new insights into the resistance mechanisms of tobacco and provide a scientific basis for addressing chilling stress challenges in practical production.

## Data Availability

The mass spectrometry proteomics data have been deposited to the ProteomeXchange Consortium (https://proteomecentral.proteomexchange.org) via the iProX partner repository with the dataset identifier PXD055372.
